# Supplementary figures and images for: How do symptoms of each joint contribute to global pain, disease activity and functional disability in rheumatoid arthritis?—A comprehensive association study using a large cohort
Source: PLoS One. 2023 Aug 25;18(8):e0285227. doi: 10.1371/journal.pone.0285227 (PMC10456163; doi:10.1371/journal.pone.0285227)

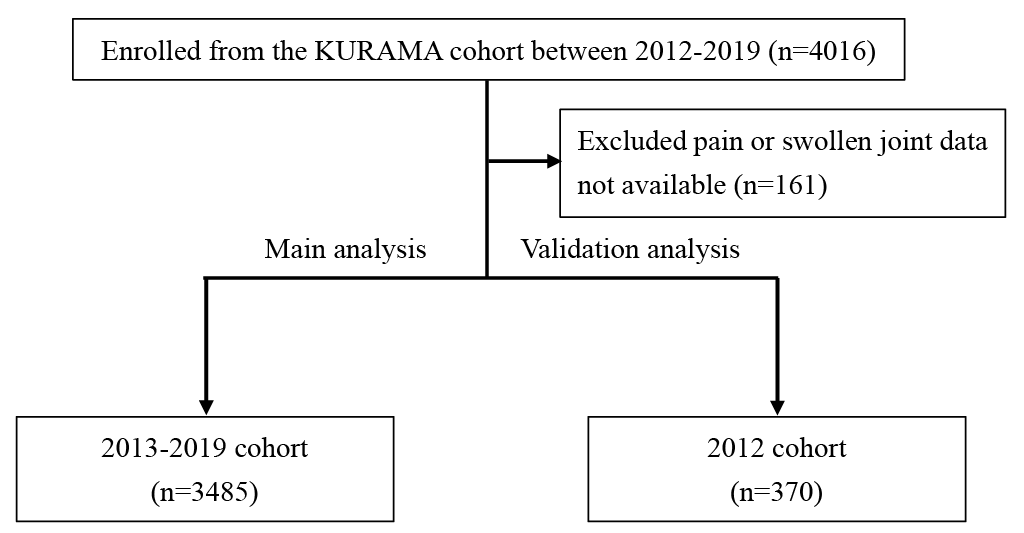

Supplement: S1 Fig — (TIFF) [file pone.0285227.s001.tiff]

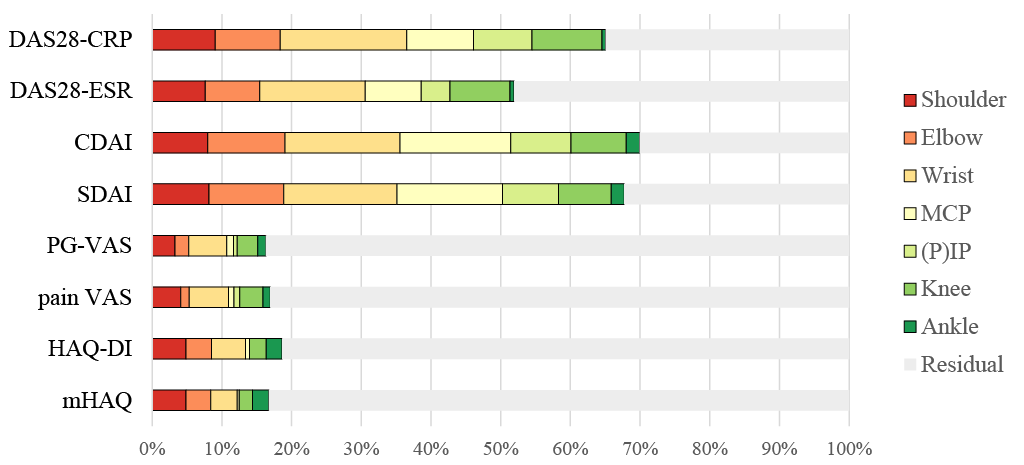

Supplement: S2 Fig — The contribution ratio of each joint is summarized in the graph. The contribution ratio was calculated using the ratio of the partial R-squared values including residual as a result of multivariate analysis. Joints whose partial R-squared values were less than 0.01 were excluded. (TIFF) [file pone.0285227.s002.tiff]

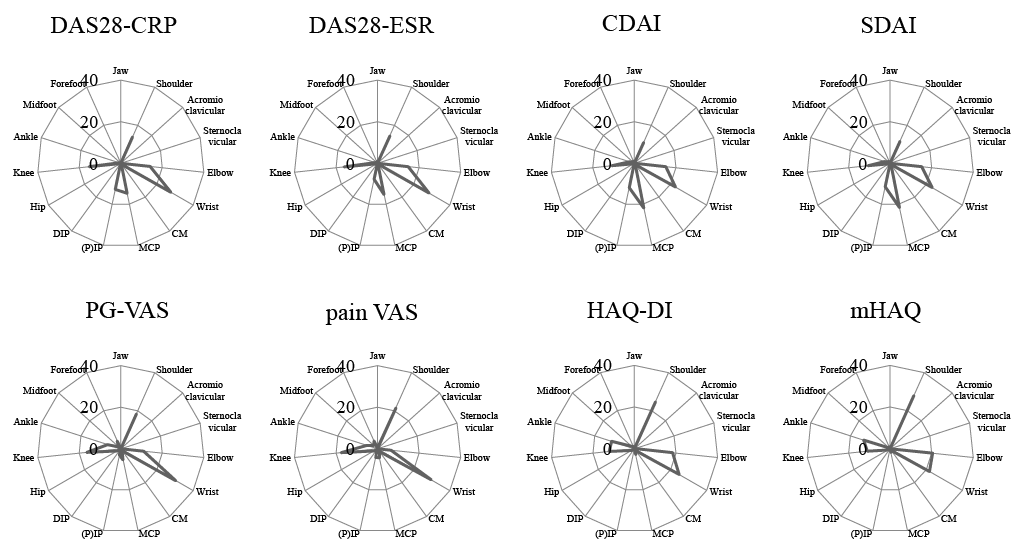

Supplement: S3 Fig — (TIFF) [file pone.0285227.s003.tiff]

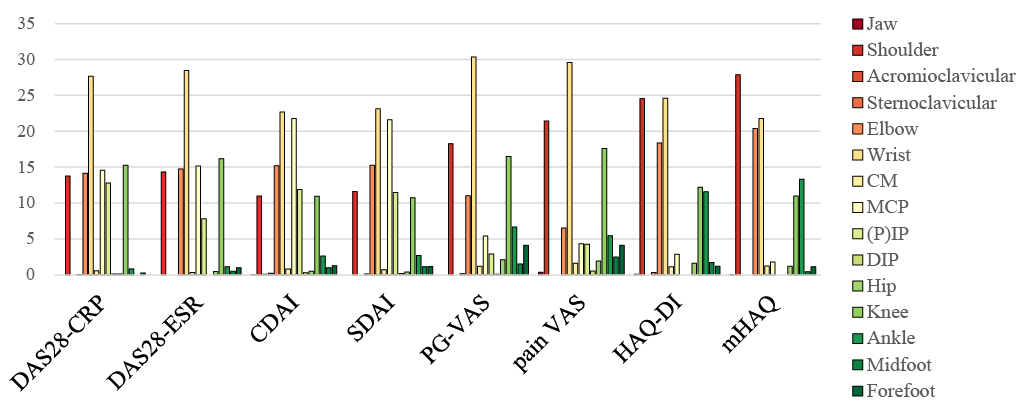

Supplement: S4 Fig — (TIFF) [file pone.0285227.s004.tiff]

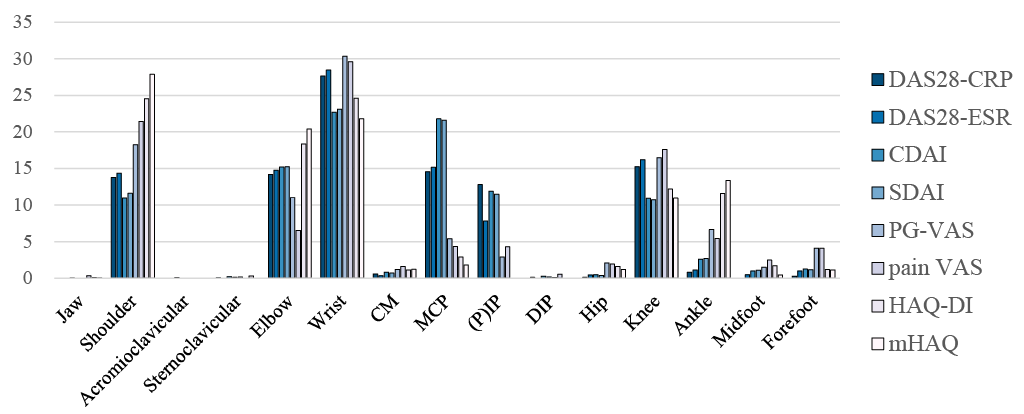

Supplement: S5 Fig — (TIFF) [file pone.0285227.s005.tiff]

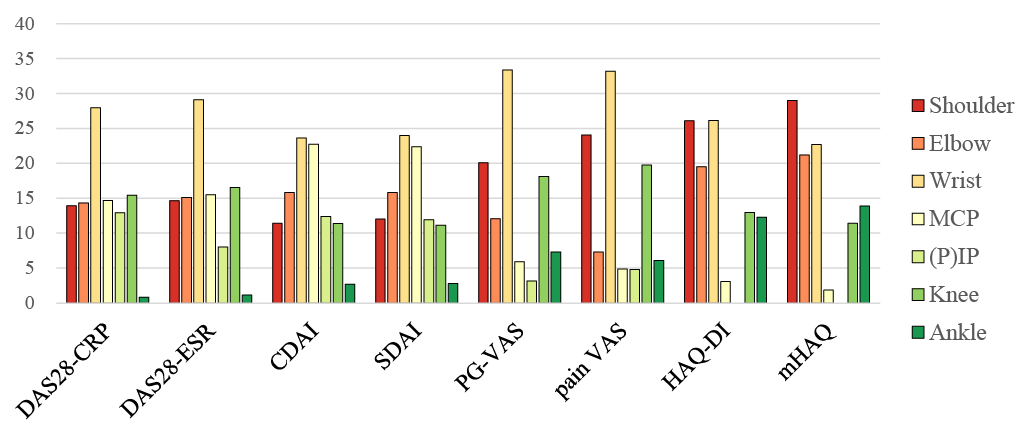

Supplement: S6 Fig — The contribution ratio of each joint is summarized in the bar chart. Joints whose partial R-squared values were less than 0.01 were excluded. (TIFF) [file pone.0285227.s006.tiff]

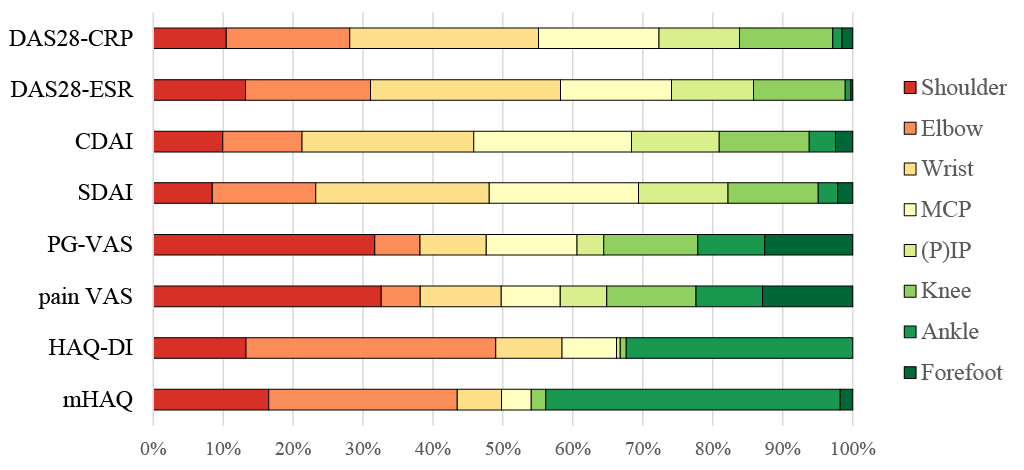

Supplement: S7 Fig — The contribution ratio of each joint is summarized in the graph. The contribution ratio was calculated using the ratio of the partial R-squared values as a result of multivariate analysis. Joints whose partial R-squared values were less than 0.01 were excluded. (TIFF) [file pone.0285227.s007.tiff]

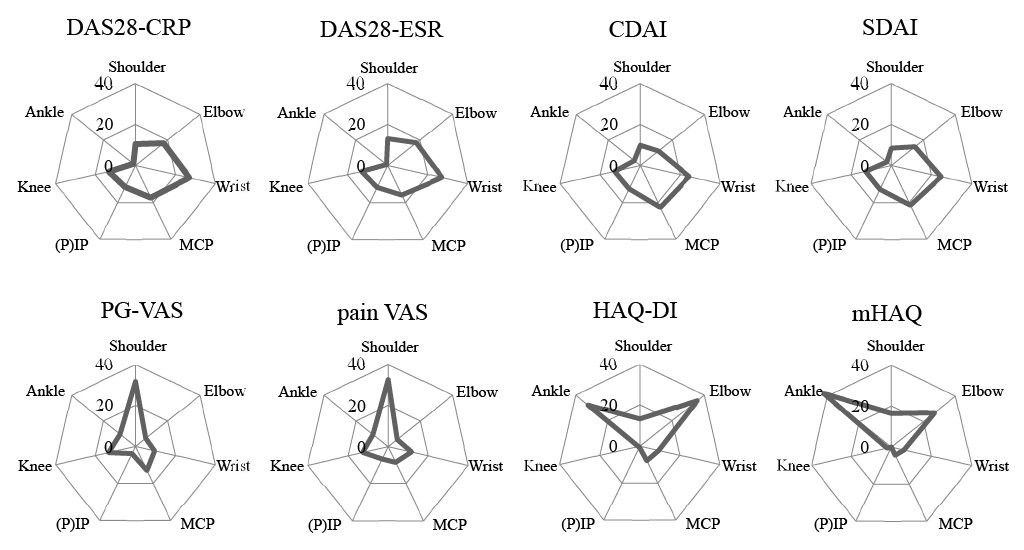

Supplement: S8 Fig — (TIFF) [file pone.0285227.s008.tiff]

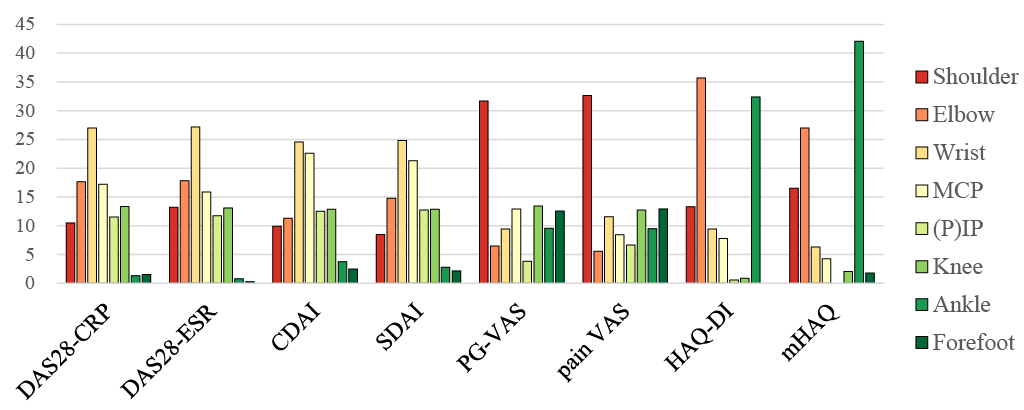

Supplement: S9 Fig — (TIFF) [file pone.0285227.s009.tiff]

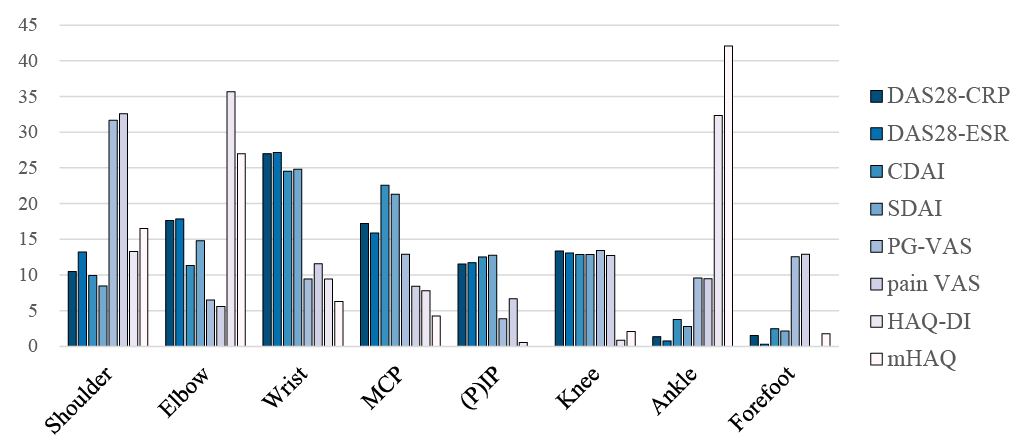

Supplement: S10 Fig — (TIFF) [file pone.0285227.s010.tiff]

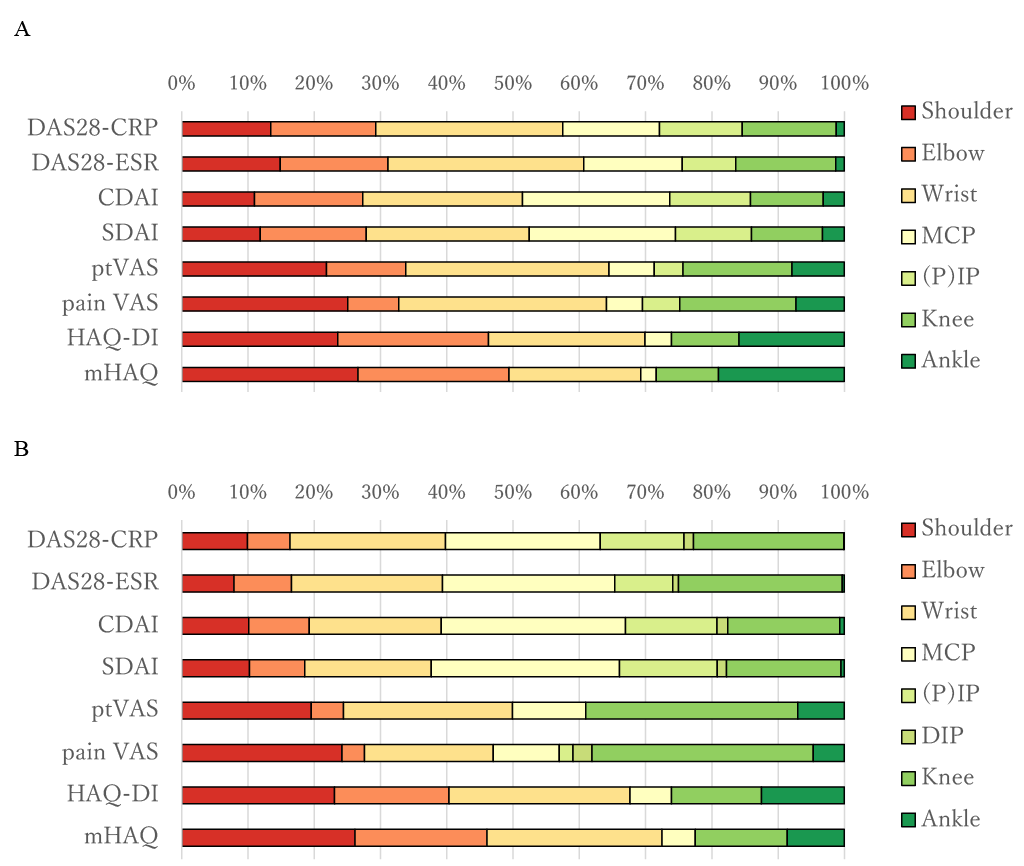

Supplement: S11 Fig — Relative contribution of various joints to DAS, DAI, VAS, and HAQ in the 2012–2018 (A) and in the 2019 (B) KURAMA cohort. (TIFF) [file pone.0285227.s011.tiff]

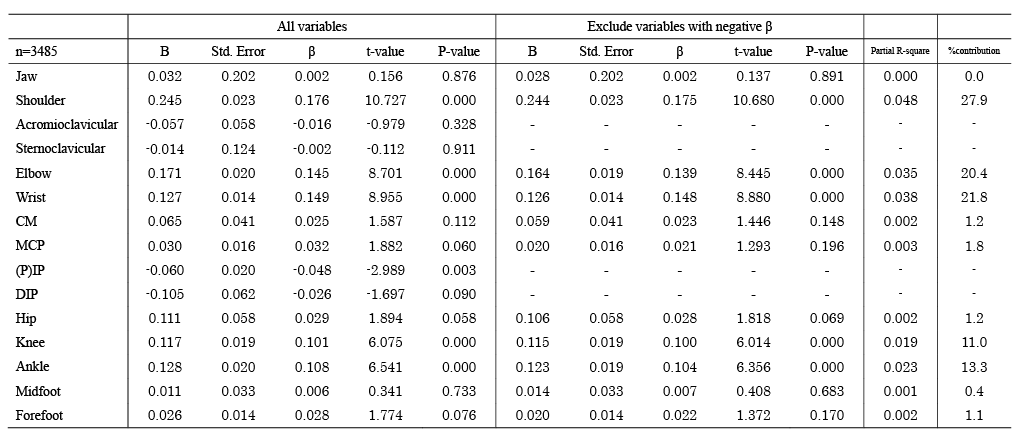

Supplement: S1 Table — 1. Multivariable association for the contribution of various joint to DAS28-ESR in the 2013–2019 KURAMA cohort. 2. Multivariable association for the contribution of various joint to CDAI in the 2013–2019 KURAMA cohort. 3. Multivariable association for the contribution of various joint to SDAI in the 2013–2019 KURAMA cohort. 4. Multivariable association for the contribution of various joint to pain VAS in the 2013–2019 KURAMA cohort. 5. Multivariable association for the contribution of various joint to mHAQ in the 2013–2019 KURAMA cohort. (ZIP) [file pone.0285227.s012.zip › S1-5_Table.tiff]

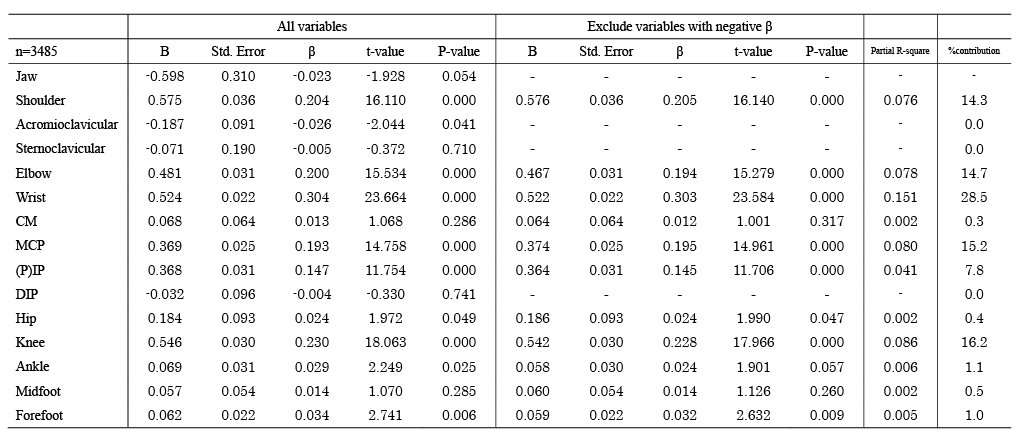

Supplement: S1 Table — 1. Multivariable association for the contribution of various joint to DAS28-ESR in the 2013–2019 KURAMA cohort. 2. Multivariable association for the contribution of various joint to CDAI in the 2013–2019 KURAMA cohort. 3. Multivariable association for the contribution of various joint to SDAI in the 2013–2019 KURAMA cohort. 4. Multivariable association for the contribution of various joint to pain VAS in the 2013–2019 KURAMA cohort. 5. Multivariable association for the contribution of various joint to mHAQ in the 2013–2019 KURAMA cohort. (ZIP) [file pone.0285227.s012.zip › S1-1_Table.tiff]

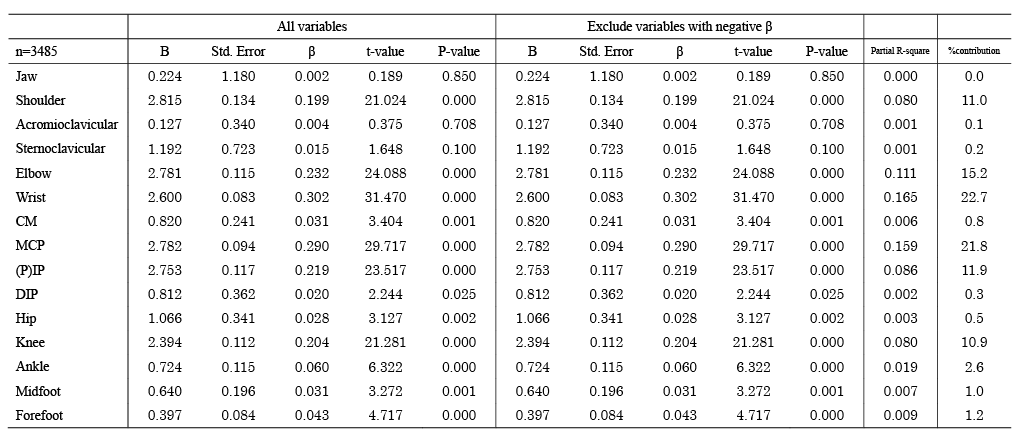

Supplement: S1 Table — 1. Multivariable association for the contribution of various joint to DAS28-ESR in the 2013–2019 KURAMA cohort. 2. Multivariable association for the contribution of various joint to CDAI in the 2013–2019 KURAMA cohort. 3. Multivariable association for the contribution of various joint to SDAI in the 2013–2019 KURAMA cohort. 4. Multivariable association for the contribution of various joint to pain VAS in the 2013–2019 KURAMA cohort. 5. Multivariable association for the contribution of various joint to mHAQ in the 2013–2019 KURAMA cohort. (ZIP) [file pone.0285227.s012.zip › S1-2_Table.tiff]

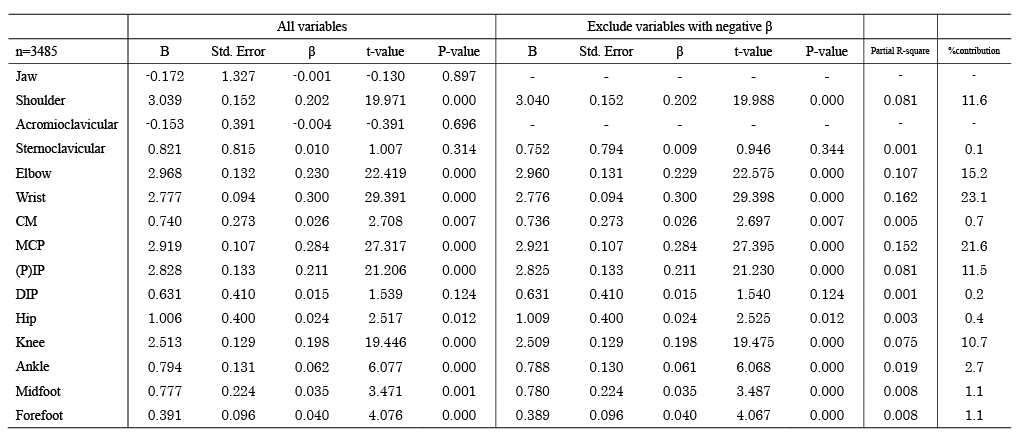

Supplement: S1 Table — 1. Multivariable association for the contribution of various joint to DAS28-ESR in the 2013–2019 KURAMA cohort. 2. Multivariable association for the contribution of various joint to CDAI in the 2013–2019 KURAMA cohort. 3. Multivariable association for the contribution of various joint to SDAI in the 2013–2019 KURAMA cohort. 4. Multivariable association for the contribution of various joint to pain VAS in the 2013–2019 KURAMA cohort. 5. Multivariable association for the contribution of various joint to mHAQ in the 2013–2019 KURAMA cohort. (ZIP) [file pone.0285227.s012.zip › S1-3_Table.tiff]

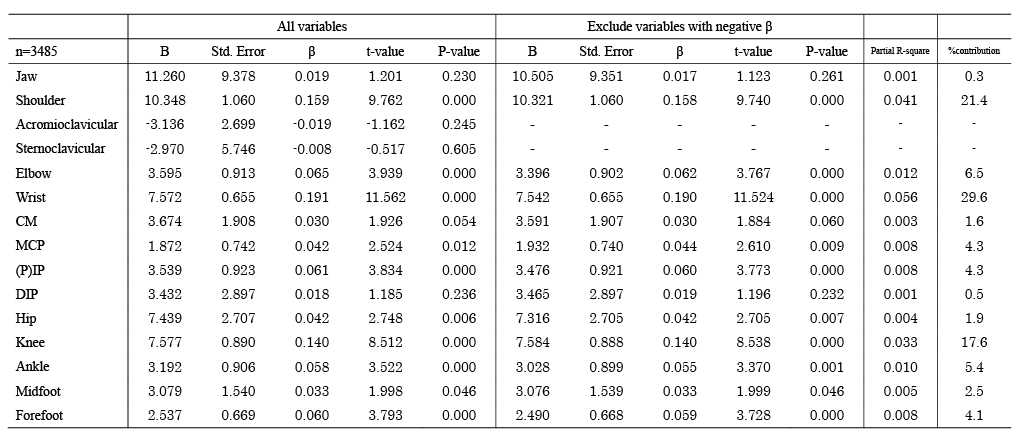

Supplement: S1 Table — 1. Multivariable association for the contribution of various joint to DAS28-ESR in the 2013–2019 KURAMA cohort. 2. Multivariable association for the contribution of various joint to CDAI in the 2013–2019 KURAMA cohort. 3. Multivariable association for the contribution of various joint to SDAI in the 2013–2019 KURAMA cohort. 4. Multivariable association for the contribution of various joint to pain VAS in the 2013–2019 KURAMA cohort. 5. Multivariable association for the contribution of various joint to mHAQ in the 2013–2019 KURAMA cohort. (ZIP) [file pone.0285227.s012.zip › S1-4_Table.tiff]
