# Supplementary material for: How do symptoms of each joint contribute to global pain, disease activity and functional disability in rheumatoid arthritis?—A comprehensive association study using a large cohort
Source: PLoS One. 2023 Aug 25;18(8):e0285227. doi: 10.1371/journal.pone.0285227 (PMC10456163; doi:10.1371/journal.pone.0285227)
Supplement: Attachment — Submitted filename: Response to Reviewers.docx [file pone.0285227.s014.docx]

**Point by point responses to the reviewers’ comments**

First of all, we sincerely appreciate your careful evaluations. The answers for the specific comments were written below each comment in bold type. Revised text is highlighted by red.

**Reviewer #1:**

*This study analyzes the joint contribution of any disease activity index for RA.
In particular, the focus on the hip and MTP joint is unique.*

**We were delighted to hear that you acknowledge the importance of this manuscript.**

*How this research can be applied or utilized in daily clinical practice is still being determined.*

**Response:**

**That is a very important point in this study. We consider that it is helpful for practitioners to consider which joint(s) should be prioritized for treatment for the particular patient, as described in line 308-316. We believe that the comparison of the results of Fig. 3 with the patient's painful and swollen joints would greatly help determine a treatment strategy.**

**Reviewer #2:**

*To examine the association between joint lesion location and disease status assessment tools, the authors performed a multivariate analysis of the relative contribution of symptoms at various joint sites to disease status assessment tools, VAS scores, and functional disability indices using data from a large Japanese RA cohort. The results showed that the disease state assessment tools was affected differently depending on the site of the symptomatic joint. I believe that this is an important study because of its relevance to daily clinical practice. I have some specific comments.*

**We deeply appreciate the reviewer’s comments which is rewarding to our commitments and efforts to this study.**

Major points:

*1. Why is it that in Table 1 the patient background is quite different in 2012 compared to 2013-2019? It may be less valid to consider the 2013-2019 registrants as a general population. Has the method of patient enrollment changed since 2013? The authors should more clearly state why they chose 2012 as the dataset for the Validation study.*

**Response:**

**The difference in patient background data between 2012 and 2013-2019 can be attributed to the gradual changes in treatment over time. Items related to disease activity are improving, PSL use is decreasing while the use of bDMARD and tsDMARD has been increasing while there are no differences in age or disease duration. Also, nothing has been changed in patient enrollment methods.**

*2. Conversely, the same analysis could be done with the 2012-2018 registrants as the main study and the 2019 registrants as the validation study. If the same results are obtained, the reliability of the results of this study will increase.*

**Response:**

**Thank you for your helpful advice. We reanalyzed the whole data using 2012-2018 as the main analysis and 2019 as validation one. Almost the same results were obtained for the main data. For 2019, the contribution of the knee and MCP joints tended to be higher overall, but the rest of the data showed approximately the similar trend (suppl Fig. 11). We added the descriptions in the results section (line 245-249).**

**Reviewer #3:**

*Introduction and Discussion sections were too long.*

**Response:**

**Thank you for your suggestion, I have removed the redundant parts in the Introduction and Discussion sections.**

*“indices” and “indexes” were used in the manuscript. Please use one of them.*

**Response:**

**Thanks for pointing that out, I have standardized on indexes.**

*In line 269, [8] would be [10].*

**Response:**

**Thank you for pointing that out. You are correct and I have corrected it to [10].**

(The end of the responses)
